# What’s in a Number: Do Transfer Rates Reflect Nursing Home Quality?

**DOI:** 10.1111/jgs.70349

**Published:** 2026-02-24

**Authors:** Debra Saliba, Vincent Mor, Kisa A. Hilliard, Amy L. Mochel, Margaret Baumann, Rebecca Boxer, Heather D’Adamo, Hiroshi Gotanda, Kim W. House, Seema Joshi, Linda Sohn, Arti Tayade, Sarah Tubbesing, Dan R. Berlowitz, Orna Intrator, Roee Gutman, Ciaran S. Phibbs, Joseph G. Ouslander

**Affiliations:** 1 GRECC and HSR Center of Innovation, Greater Los Angeles Veterans Administration Health System, Los Angeles, California, USA; 2 Borun Center, University of California Los Angeles, Los Angeles, California, USA; 3 RAND Health, Santa Monica, California, USA; 4 Department of Health Services, Policy & Practice, Brown University School of Public Health, Providence, Rhode Island, USA; 5 Center of Innovation, Providence Veterans Administration Medical Center, Providence, Rhode Island, USA; 6 Jesse Brown VA Geriatrics & Extended Care, Chicago, Illinois, USA; 7 Department of Medicine, University of Illinois, Chicago, Illinois, USA; 8 Department of Medicine, University of California Davis, Sacramento, California, USA; 9 Department of Veterans Affairs Greater Los Angeles Healthcare System, Geriatrics and Extended Care, Los Angeles, California, USA; 10 Department of Medicine, Cedars- Sinai Medical Center, Los Angeles, California, USA; 11 Joseph Maxwell Cleland VA Medical Center, Atlanta, Georgia, USA; 12 Department of Medicine, Emory University School of Medicine, Atlanta, Georgia, USA; 13 Dwight D. Eisenhower VA Medical Center, Leavenworth, Kansas, USA; 14 Colmery O’neil VA Medical Center, Topeka, Kansas, USA; 15 VA Puget Sound Health Care System, University of Washington, Seattle, Washington, USA; 16 Department of Public Health, University of Massachusetts Lowell, Lowell, Massachusetts, USA; 17 James J. Peters VA Medical Center, Bronx, New York,USA; 18 Brookdale Department of Geriatrics & Palliative Medicine, Icahn School of Medicine at Mount Sinai, New York, New York, USA; 19 Department of Biostatistics, Brown University School of Public Health, Providence, Rhode Island, USA; 20 Palo Alto Veterans Administration Medical Center, Palo Alto, California, USA; 21 Department of Pediatrics, Stanford University School of Medicine, Palo Alto, California, USA; 22 Florida Atlantic University Schmidt College of Medicine, Boca Raton, Florida, USA

**Keywords:** appropriateness, hospitalizations, nursing homes, quality, transfers, veterans administration

## Abstract

**Background:**

Hospitalization rates from nursing homes (NHs) have gained traction as pragmatic quality measures that can be derived from claims data. However, claims-based hospitalization measures do not account for clinical complexity and the extent to which they reflect quality of care or quality of transfer decision making is unknown. We aim to examine agreement between a claims-based measure of potentially avoidable hospitalizations and expert clinician review of transfer decision making and care quality.

**Methods:**

We randomly selected 252 hospital transfers across eight Veterans Administration (VA) NHs, known as Community Living Centers (CLCs). Eleven expert clinicians independently completed Structured Implicit Reviews (SIRs) of medical records to assess: (1) whether the transfer decision was appropriate (i.e., hospital was the lowest safe level-of-care given the resident’s acute condition); (2) quality of care for evaluation or treatment of the acute change (adequate management of acute change), (3) quality of care for chronic conditions and preventing decline. We used VA Corporate Data Warehouse (CDW) data to determine a claims-based measure of potentially avoidable hospitalization.

**Results:**

CDW data were available for 242 VA hospitalizations. The claims-based measure categorized 29 (12%) hospitalizations as potentially avoidable; only 2 of which matched the 20 SIR identified as inappropriate decisions to transfer. Furthermore, the claims-based measure flagged only 5 of 33 cases rated as inadequate treatment of acute decline and 6 of 17 rated as poor quality of chronic disease or preventive care.

**Conclusions:**

In a geographically diverse sample of CLC transfers, independent clinical experts’ judgments of transfer decision appropriateness, quality of care for acute decline, and quality of chronic care differ from a claims-based potentially avoidable hospitalizations measure. Findings underscore the need for nuanced clinical consideration of hospitalization metrics for assessing quality and for understanding which aspects of care should be addressed to safely reduce NH transfers to hospitals.

## 1 | Introduction

Transfer of nursing home (NH) residents to emergency departments (EDs) and hospitals may signal potentially poor NH care quality [1], expose residents to risks [2, 3], and can be an inefficient use of resources [4, 5]. However, transfer may be an appropriate response to resident needs that require ED or hospital level care [1]. Distinguishing NHs with excessive transfers from those with better prevention and on-site management of acute changes in condition has significant implications for residents, providers, and health systems [6], in addition to having important ramifications for quality improvement [7, 8], quality metrics [9], and facility payments [10, 11].

Concern exists as to whether transfer rates alone capture the complexities of decision making and medical management in this medically complex population [12–14] at increased risk for diseasedisease and drug-disease interactions [15–17]. The actual transfer decision ideally involves carefully assessing the match between resident need and the NH’s ability to meet that need. The latter includes considering capacity to assess the acute change in condition, communicate changes among the care team, develop and implement management plans, and monitor resident response.

In addition to the quality of transfer decisions, transfer rates may signal that poor quality of preventive care or chronic disease management contributed to the deterioration driving transfer. It is also possible that errors in the recognition of and management of the acute decline contributed to the severity of decline that resulted in the need for transfers. Once again, the medical complexity of the population challenges a fixed presumption of poor care. A resident may experience decline despite high quality of preventive care or evidence-based chronic disease management or despite prompt recognition of and appropriate efforts to manage acute change.

The complexity described above presents challenges in identifying rubrics for monitoring hospitalizations of NH residents. An all-cause hospitalization metric may signal a problem somewhere in nursing home care but may fail to adequately capture clinical complexity. Potentially avoidable or preventable hospitalization measures have been developed as a subset of the all-cause hospitalization measure in an effort to focus on more actionable conditions likely to reflect quality of care [5, 6, 8, 18–23]. Publicly reported potentially avoidable hospitalization measures, like all-cause hospitalizations, are determined from pre-existing hospital claims data. The ability to leverage already collected data from a source external to the NH is viewed as a pragmatic strength of a claims-based measure of potentially avoidable hospitalizations. Despite the intent to provide a more salient measure, claims-based potentially avoidable hospitalizations metrics may not account for the NH environment [13, 24, 25], and the available administrative data elements may fail to capture the complexities and interactions faced by providers when making transfer decisions [26]. As such, published hospitalization rates may not directly reflect the quality of transfer decisions or other aspects of care quality, may unfairly penalize some providers, and could create perverse incentives that undermine the integrity of clinical decision making [27].

One alternative to reliance on overall and diagnosis-based rates for the identification of potential problems with transfers to EDs and hospitals is implicit, that is, inferred, quality review of the medical record by an expert clinician. Many quality review committees accept clinician review as a “gold-standard.” However, unguided implicit review can be unreliable, varying widely in determinations made on the same case by different reviewers [28].

An alternative approach that allows consideration of clinical complexity while also achieving good interrater agreement is the Structured Implicit Review. Structured Implicit Review guides reviewers in systematically assessing components of decisions before rendering final assessments of appropriateness and quality. This approach is particularly well-suited for review of clinical care in heterogeneous and complex populations such as those found in NHs. Structured Implicit Review has been shown to produce reliable assessments of the appropriateness of decisions to transfer NH residents to EDs and hospitals [1].

The current study compares assessments of NH performance using different approaches to examining transfers. We compare a commonly used claims-based potentially avoidable hospitalizations measure to expert clinician Structured Implicit Review of transfer decision appropriateness, quality of care for managing the acute change, and the quality of care for chronic conditions and prevention for randomly selected transfers from eight VA Community Living Centers (CLCs) to VA hospitals and EDs.

## 2 | Methods

### 2.1 | Study Overview

As part of a larger study testing the Interventions to Reduce Acute Care Transfers (INTERACT) [5, 8] program in eight VA CLCs [23, 29, 30], 11 trained, expert clinicians completed Structured Implicit Review of 252 transfers from CLCs to EDs and hospitals to determine: (1) whether, given the resident’s condition at the time of transfer, the resident could have been safely treated at a lower-level-of-care than the ED or hospital (i.e., decision to transfer was not appropriate); (2) the quality of care for the acute change prior to transfer; (3) the quality of care for prevention or management of conditions to avoid acute decline, and (4) the potential importance of better quality for reducing transfers (i.e., potential alterability).

### 2.2 | Conceptual Framework

**FIGURE 1 |:**
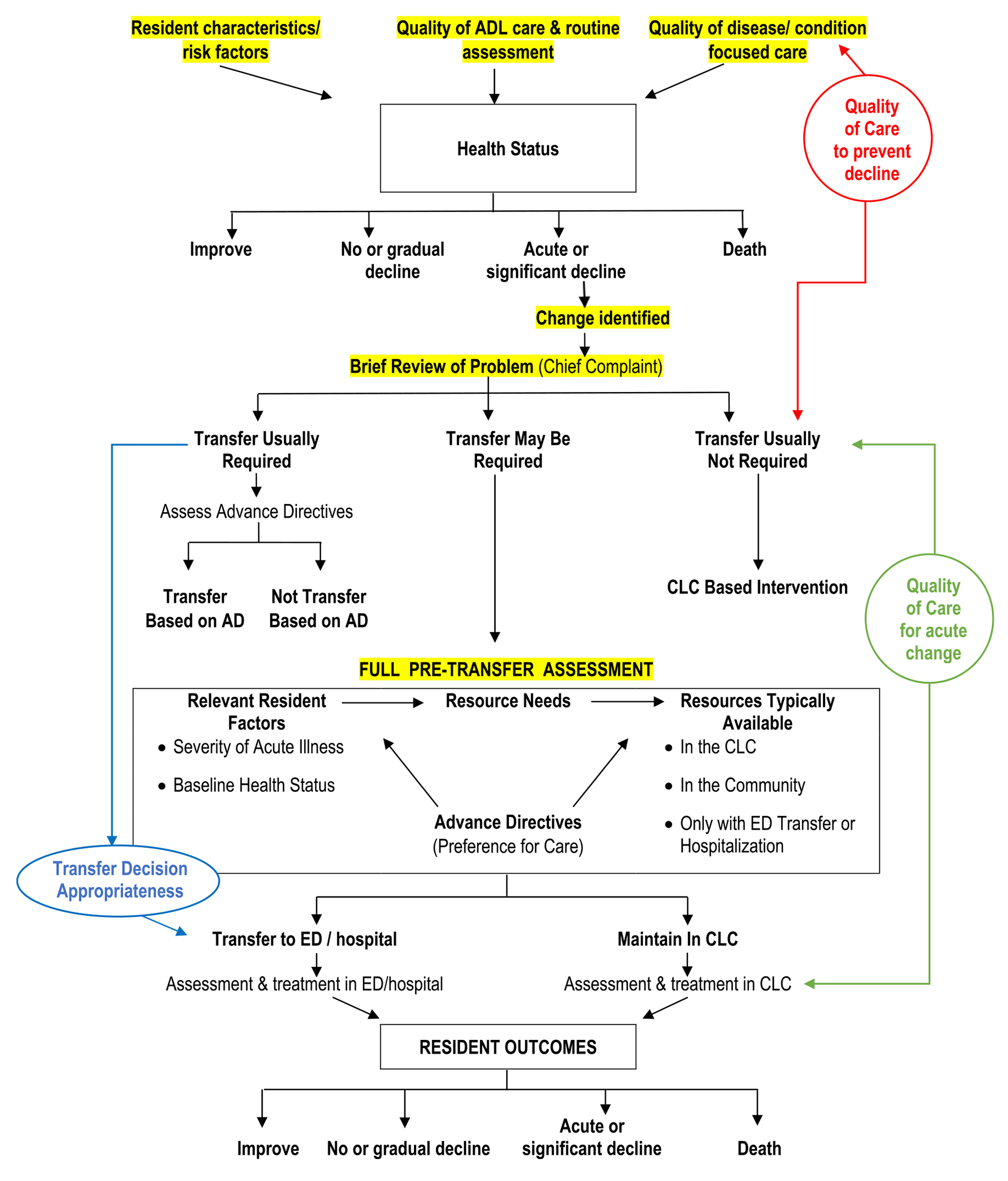
Nursing home to hospital transfers: overall algorithm.

The Structured Implicit Review (SIR) for transfer decisions’ conceptual framework (Figure 1), based on the literature and expert clinicians’ input, outlines relevant elements for decision making. The SIR form includes sequential questions intended to ensure that each reviewer systematically considers the same clinical data elements before rendering summary judgments about care quality and transfer appropriateness [1]. Accompanying instructions, designed to ensure consistent review of specific data sources in medical records, create shared understanding across reviewers about the questions and response options.

Each reviewer uses clinical judgment to synthesize data and answer questions about the resident’s baseline health status and preferences, acute change and related needs, care provided, and response to care. The reviewer then makes summary determinations about: (1) whether the transfer or admission decision was appropriate (i.e., the ED or hospital was the lowest safe level of care given the resident’s acute change in condition); (2) better quality of NH care could have reversed or stabilized the acute change (inadequate treatment of acute change), (3) the quality of care provided by the NH to manage chronic conditions and provided preventive care to avoid the acute change, and (4) whether improvements in quality could potentially reduce transfer need (potential alterability) opportunities for continuous process improvement. Reviewers rate ED transfer decisions separately from hospitalization decisions, because available information for the same patient varies between ED and hospital records and separating the events therefore improves agreement for ratings.

### 2.3 | Reviewer Training and Percent Agreement

We recruited 11 reviewers (10 geriatricians, 1 advanced practice nurse) for their clinical expertise within VA CLCs. To avoid bias, we did not recruit reviewers from the intervention sites and did not give reviewers specific information about the INTERACT pragmatic trial.

We used an iterative training approach associated with enhanced agreement in other studies [1, 31, 32]. Prior to in-person training, each reviewer received the SIR instruction manual, an SIR form, and a medical record to practice completing SIR. Reviewers then convened for a 1.5-day in-person training that included an in-depth review of SIR instructions and discussion of reviewers’ SIR answers for the pre-assigned training case. Reviewers then independently reviewed a second transfer record, followed by a discussion of their responses to all SIR questions for the 2nd case. After the training session, each reviewer independently reviewed two additional training records during the subsequent month. We analyzed responses and discussed item disagreement during a follow-up call with reviewers.

We then assigned five common charts for independent review by all reviewers and analyzed percent agreement. Across these five common records, reviewers achieved a 96% level of agreement with respect to judging the appropriateness of the transfer decision, 94% for whether inadequate treatment of acute change contributed to transfers’ need, 77% for assessment of the quality of NH care for managing chronic disease and providing preventive care. Disagreements in responses were discussed in a group follow-up call.

### 2.4 | SIR Sample and Record Review

The sample was identified based on CLC residents admitted to VA hospitals. We excluded “planned hospitalizations” using a commonly applied algorithm to identify codes for procedures that are typically planned (e.g., scheduled surgeries, chemotherapy) [18] before drawing a random sample of 253 unplanned hospitalizations from the experimental CLCs (124 prior to and 129 during the intervention period). We combined the pre and post cases before randomly assigning cases across the 11 reviewers, with each assigned 23. We ensured the distribution of each facility’s cases across reviewers. One assigned case was dropped after record review revealed it was a planned transfer missed by the planned hospitalization algorithm, resulting in a sample of 252 hospital transfers.

Reviewers examined the resident’s CLC record, ED record (where available), and first 3 days in the hospital.

The project was approved by the central VA-IRB. Each reviewer was trained in and signed a confidentiality and privacy agreement. We assigned records to reviewers in the VA Electronic Health Records system (VISTAWeb). Reviewers entered their SIR responses on an online secure data form.

Results were analyzed in Excel. To assess the appropriateness of the hospitalization decision, we categorized reviewer responses to the question asking whether the hospital was the lowest safe level of care to meet resident needs, honoring the Veterans level of care directives, as Yes (definitely or probably) vs. No (definitely or probably). Response options for whether poor quality of care for the acute change contributed to transfer need (inadequate evaluation or management of acute change) were Yes vs. No. Ratings for quality of care for prevention or avoidance of acute decline were categorized as Poor (poor or very poor), fair, and good (excellent or good). The potential importance of quality improvement for reducing transfer need (opportunities for continuous process improvement) was categorized as important (very important or somewhat important) vs. not important (not very important or not at all important).

### 2.5 | Hospitalization Rates Based on Administrative Data

For the claims-based measure of potentially avoidable hospitalization, we applied an algorithm based on the Agency for Healthcare Research and Quality’s Prevention Quality Indicator (AHRQ-PQI), a commonly reported metric for potentially avoidable hospitalizations from NHs [5, 6, 8, 18–20, 23]. The AHRQ-PQI was initially developed to use hospital admission diagnoses to identify ambulatory care sensitive conditions and provide a single summative rate of potentially avoidable hospitalization that captures the adequacy of access to appropriate outpatient care to: manage acute conditions at early stages, manage chronic disease, and provide preventive services. These conditions were later applied for use in measuring NH transfers.

For the current study, claims-based transfer rates were limited to VA data obtained from the VA Corporate Data Warehouse, creating better alignment with SIR review of VA CLC, ED, and hospital medical records. For each hospitalization in the pre-intervention and concurrent intervention samples, we determined whether, based upon the discharge diagnoses and primary reason for hospital admission, that hospitalization met the AHRQ-PQI criteria of a potentially avoidable hospitalization.

## 3 | Results

### 3.1 | Rating the Appropriateness of ED Transfer Decision and Hospital Admission Decision

The random sample of cases included 67 cases transferred to the ED prior to hospitalization in the pre-intervention period and 59 in the intervention period. Reviewers deemed ED transfer decision to be inappropriate for 5 (7.5%) pre-intervention and 9 (15.3%) during the intervention period. Table 1 shows reviewer assessments of hospitalizations. The hospitalization decision was deemed inappropriate (i.e., the resident could have been safely treated at a lower-level-of-care) for 9 of 123 cases in the pre-intervention sample (7.3%) and 15 of 129 (11.6%) in the intervention sample (Chi-square test for pre-intervention and intervention difference for both ED and hospital = 0.24).

### 3.2 | Rating Quality of Care for Evaluation or Management of Acute Change

Reviewers concluded that poor or inadequate quality of care for the acute change contributed to the need to transfer for *n = 16* pre-intervention cases (13%) and *n = 19* (14.7%) of post-intervention cases.

### 3.3 | Rating CLC Quality of Care for Prevention or Management of Chronic Conditions to Prevent Acute Decline or Deterioration

Reviewers assessed quality of care in the CLC as poor or very poor at essentially the same level for pre-intervention *n* = 10 cases (8.1%) and intervention *n* = 10 cases (7.8%). Past testing of response options for this quality assessment supported the inclusion of a “fair” rating response between very poor/poor and good/excellent ratings of prevention and chronic condition management. In the current sample, reviewers rated CLC care “fair” for 24 residents (19.5%) in the pre-intervention and 17 (13.2%) in the intervention sample.

### 3.4 | Comparing Clinician SIR to AHRQ-PQI Findings

The AHRQ-PQI algorithm could not be applied to 10 SIR cases because all required variables were not available in CDW data. We therefore limited the comparison of SIR findings to claims-based findings to a sample of 242 combined pre and post intervention cases. Table 2 shows the overlap of AHRQ- PQI PAH cases to SIR ratings for the 242 cases being compared. Of the 242 cases, the AHRQ algorithm categorized 29 (12%) as potentially avoidable hospitalizations (PAH). Only 2 of the cases that the claims-based measure flagged as potentially avoidable matched the 20 SIR cases reviewers rated as inappropriate decisions to transfer. Furthermore, the claims- based algorithm flagged only 6 (35%) of the 17 SIR reviewed cases rated as receiving poor quality of CLC preventive or chronic disease care and 8 of the 39 (20%) in which reviewers rated chronic disease or preventive care as fair.

## 4 | Discussion

In this randomly drawn sample of transfers from CLCs participating in an intervention to reduce hospitalizations, we find limited correspondence between clinical reviewers’ detailed assessment of various aspects of quality and a claims-based potentially avoidable hospitalization metric applied to these same hospitalizations. Claims-based metrics that rely on readily available administrative data are a straightforward approach for flagging facilities with possible excessive transfers. The current study, however, illustrates that claims-based metrics might not be sufficient if policymakers and healthcare institutions are aiming to safely and judiciously reduce the hospitalization of NH residents.

This is important for several reasons. It provides empirical support for the contention that although more pragmatic claims-based metrics may provide a signal of care quality, they may fall short of matching the complexity of decision making in NH populations and other clinically complex older adult populations [33]. In addition, claims-based measures may be fraught with limitations in highlighting where NH care falls short. Numerous contextual factors may drive decision making in actual care, and SIR allows reviewers to account for these. A hospital admission diagnosis of heart failure or urinary tract infection does not provide insights into whether the deterioration driving admission could have been treated outside the hospital given diagnostic uncertainty, other conditions, and needs. The findings also highlight the importance of requiring precision in interpreting and describing claims-based metrics.

These distinctions are more than semantic. Without clear agreement on the definition of “avoidable” hospitalizations, it is difficult to accurately assess the magnitude of the problem. Equally, if not more important, the lack of clarity in defining the target outcome can impede needs assessment and intervention design. Through careful case review that accounts for clinical complexity, the current study finds that in this sample of facilities, given a resident2019;s needs, a minority of hospitalizations were associated with an inappropriate decision to transfer. Likewise, a minority of transfers were deemed to be associated with poor quality of CLC care for the acute decline once it occurred. Thus, an intervention or payment approach focused on management of acute change once it occurred would likely have limited effect.

Reviewers also evaluated the quality of care to manage baseline chronic conditions and provide other preventive care and rated a minority of CLC care as poor or very poor. The reviewers rated CLC quality of preventive care and chronic condition management as only fair for more of the transfers. If poor, very poor, and fair care were combined, this might point toward more quality improvement opportunities in chronic disease management and prevention; although, even combined, these findings would apply to less than a fourth of hospitalizations.

In the current study, the in-depth SIR affords insights into where quality improvement (QI) efforts might have greater effect. The CLC-adapted INTERACT placed heavy emphasis on the communication around significant acute change in condition and root-cause analysis of transfer decisions. This included integrating the SBAR communication tool into the electronic medical record (EMR). However, INTERACT’s chronic condition algorithms and educational clinical decision support tools that might more directly address care quality contributing to the decline itself were not integrated into EMR or workflows.

Expert clinician review deemed relatively few transfer decisions to be inappropriate. Reviewers also identified a minority of transfers as having had inadequate management of the acute change in the facility and a limited percent of the acute decline events as being associated with poor quality of preventive and chronic disease management. These findings would not have been predicted based on all-cause hospitalization rates for the CLCs prior to the intervention, which were higher than national averages in community NHs [23, 34].

Notably, from a QI perspective, addressing chronic disease management and prevention would likely require more extensive educational efforts as well as changes in clinical workflows. This distinction is consistent with a recent demonstration project in community NHs that showed no effect from a model providing facility and provider payments for treating residents with select acute conditions within facilities. That project’s evaluation found that a focus only on payments for specific acute conditions did not support facilities in making needed staffing and workflow changes [35].

SIR, like many clinical assessments, requires reviewer training. Additionally, full, systematic review of medical records to answer formative questions requires, on average, about 1 h per case. Thus, SIR is less suited for generating public quality report cards; rather, its utility lies in its ability to consider care complexities and support deeper understanding of quantitative transfer rates as an important step in targeting which phase(s) of care warrant deeper QI assessment. It also reminds us to view rates as a less-than-definitive signal of problematic transfer decisions.

This study has limitations that should be considered in inter-preting the findings. Most importantly, case reviews were limited to transferred residents. We did not assess whether transfers were being under-utilized among CLC residents. Additionally, although reviewer agreement for determining the appropriateness of the transfer decision and the quality of care for the acute condition was extremely high (96% and 94% respectively), percent agreement in rating the quality of preventive care and chronic disease management was lower at 77%. This range in agreement reflects the primary focus of the SIR assessment on the appropriateness of the transfer decision and care being provided for acute decline. Additionally, determining whether poor care may have led to the condition that required hospital transfer is more complicated since reviewers had to make causal attributions and consider more data points over time.

This close examination by clinical experts of a geographically diverse sample of transfers from multiple VA CLCs shows that appropriate transfer decisions, potentially avoidable hospitalizations, and prevention of decline have different meanings when applied to a hospital transfer from a NH. These findings underscore the need for more serious and nuanced clinical consideration of the meaning of claims-based hospitalization and re-hospitalization metrics. The common practice of using these labels interchangeably undermines the opportunity to better understand what aspects of care should be addressed in efforts to successfully and safely reduce hospital transfers from nursing homes.

## Figures and Tables

**TABLE 1 |:** Results of structured implicit review of hospitalizations.

Assessment	Pre		Post		Total	
	*n* = 123		*n* = 129		*n* = 252	
	n	%	n	%	n	%
Transfer decision not appropriate^a^	9	7.3	15	11.6	24	9.5
Inadequate quality of acute care in CLC	16	13	19	14.7	35	13.9
Quality of CLC care to prevent acute decline^b^	10	8.1	10	7.8	20	7.9
Poor	10	8.1	10	7.8	20	7.9
Fair	24	19.5	17	13.2	41	16.3

a Hospital was NOT the lowest level of care where the Veteran’s needs could be safely met.

b Rating of quality of care provided by CLC staff categorized as poor (poor or very poor), fair, or good (excellent or good).

**TABLE 2 |:** Comparison of structured implicit review (SIR) ratings to claims-based potentially avoidable hospitalization measure.

Rating	Total			
	*n* = 242		Overlap with claims- based avoidable^a^	
	n	%	%	
Claims-based: Potentially Avoidable Hospitalization	29	12		
SIR: Transfer Decision Not Appropriate^b^	20	8.3	2	10
SIR: Quality of care for acute change inadequate	33	13.6	5	15.2
SIR: Quality of chronic disease or preventive care poor^c^	17	7	6	35.3
SIR: Quality of chronic disease or preventive care fair	39	16	8	20

a Number and % of each SIR rating as potentially quality problem also identified as avoidable by claims-based potentially avoidable hospitalization algorithm.

b Hospital was NOT the lowest level of care where the Veteran's needs could be safely met.

c Quality of chronic disease management and other preventive care provided by CLC was poor or very poor.

## Summary

- Key points
  - Emergency department and hospital services may sometimes be needed to provide nursing home residents with goal concordant care. However, a proportion of this care could be managed appropriately without hospital transfer and the associated health risks and costs.
  - Quality improvement and cost saving efforts have focused on reducing the rates of nursing home residents’ emergency department visits and hospital stays. The transfers targeted for reduction have been variably labeled “potentially avoidable,” “inappropriate,” “preventable,” and “unnecessary” in the literature, which uses these labels interchangeably.
  - The current investigation uses trained and guided expert clinician review that accounts for clinical complexity to show that rates and resulting implications vary depending on the label and associated definition applied.
- Why does this paper matter?
  - Effective data-driven evaluation requires clarity and understanding of the data and metrics being used. The morbidity and costs associated with the transfer of nursing home residents to hospitals and emergency departments have focused attention on measuring nursing home performance related to these transfers. To monitor transfers and identify best strategies for improving related nursing home care, it is important to distinguish between potentially avoidable hospitalizations estimated from diagnoses in hospital claims and expert clinician assessment of inappropriate transfer decisions, inadequate management of acute change, and poor quality of chronic disease and preventive care. The current study supports precise terminology to facilitate shared understanding of these events and the development of strategies, incentives, and disincentives to safely reduce nursing home transfers to hospitals and thereby reduce complications of hospitalization and their related costs.
